# Network structure from a characterization of interactions in complex systems

**DOI:** 10.1038/s41598-022-14397-2

**Published:** 2022-07-11

**Authors:** Thorsten Rings, Timo Bröhl, Klaus Lehnertz

**Affiliations:** 1 Department of Epileptology, University Hospital Bonn, Venusberg Campus 1, 53127 Bonn, Germany; 2 Helmholtz-Institute for Radiation and Nuclear Physics, University of Bonn, Nussallee 14-16, 53115 Bonn, Germany; 3 Interdisciplinary Center for Complex Systems, University of Bonn, Brühler Straße 7, 53175 Bonn, Germany

**Keywords:** Complex networks, Nonlinear phenomena

## Abstract

Many natural and man-made complex dynamical systems can be represented by networks with vertices representing system units and edges the coupling between vertices. If edges of such a structural network are inaccessible, a widely used approach is to identify them with interactions between vertices, thereby setting up a functional network. However, it is an unsolved issue if and to what extent important properties of a functional network on the global and the local scale match those of the corresponding structural network. We address this issue by deriving functional networks from characterizing interactions in paradigmatic oscillator networks with widely-used time-series-analysis techniques for various factors that alter the collective network dynamics. Surprisingly, we find that particularly key constituents of functional networks—as identified with betweenness and eigenvector centrality—coincide with ground truth to a high degree, while global topological and spectral properties—clustering coefficient, average shortest path length, assortativity, and synchronizability—clearly deviate. We obtain similar concurrences for an empirical network. Our findings are of relevance for various scientific fields and call for conceptual and methodological refinements to further our understanding of the relationship between structure and function of complex dynamical systems.

## Introduction

Over the years, network theory has gained recognition as a powerful tool for investigating complex dynamical systems in diverse areas of science including physics, earth and climate sciences, sociology, quantitative finance, biology, and the neurosciences^1–12^. Assuming that a system can be decomposed into subsystems or units, the system then can be described by a network of vertices—representing the units—and edges—representing couplings between units. Such a network—in the following referred to as structural network—can then be investigated using methods from graph theory that reveal information about its organization by means of characterizing topological and spectral properties as well as key constituents.

In many natural and man-made complex dynamical systems, access to couplings might be limited or even impossible. It is assumed, that in such cases, the aforementioned description of a system via a network is still possible when considering network edges as interactions between units. In order to derive such a functional network, interactions need to be characterized. This can either be achieved by some active perturbation experiments or by estimating properties of interactions from passive observations of the units’ dynamics. This ansatz has been applied, e.g., in the study of (functional) brain networks^3^, climate networks^4, 13^, protein–protein interactions^14^, gene interactions^15^, plant–pollinator interactions^16, 17^, food-webs^18^, or communication and social networks^19, 20^.

Properties of interactions—strength, direction, and (under some restrictive prerequisites) even the functional form of interactions—can be derived by fitting parameters of appropriate models to data (often with perturbation approaches) and to extract interaction-related parameters from the fits. When lacking an appropriate model, another way to estimate properties of interactions is to make use of one of various bivariate time-series-analysis techniques (based on, e.g., statistics, synchronization theory, information theory, or statistical physics; for an overview see Refs.^21–28^) applied to time series data. Then, revealing the structural network from a functional network can be regarded an inverse problem, which might not have a unique solution.

Multiple previous studies investigated to what extent structural networks can be reconstructed from data when utilizing modeling approaches^29–52^ or time-series-analysis techniques^53–63^. Most studies evaluated whether the presence or absence of edges in the structural network could be correctly identified from properties of interactions—usually by applying some threshold to these properties. Since there are by now no commonly accepted criteria for how to choose a threshold^10, 64–67^, most approaches evaluate correct identifications over a range of threshold values. Some modeling-based studies^30–32, 36, 39, 42, 48, 63^ assessed correct identifications by the relative or absolute differences between estimated model parameters and parameters of some simulated networked dynamics.

Studies predominantly reported a high—but not perfect—success in identifying edges. However, since failure to correctly identify even a single edge can drastically alter the appearance of a structural network (topologically, the difference between, e.g., a line and a ring of coupled units is just one edge), it remains unclear if the studied structural networks and the ones derived from data had a similar organization. In light of functional networks being used to characterize systems in nature^68–73^, this ambiguity (and possible concomitant dissimilarities) might prove problematical in various situations.

Addressing this issue, we investigate if and to what extent the organization of a structural network—representing a complex dynamical system—can be revealed from interactions estimated from time series of the system’s dynamics using the functional network ansatz. Specifically, we examine whether a functional network carries the same information about this organization in well-known aspects of networks^74^ on different scales. These aspects can be characterized on the global scale with clustering coefficient, average shortest path length, assortativity, as well as synchronizability and on the local scale with vertex and edge centrality measures that allow one to identify the key constituents of a network.

For the purpose of our investigation, we simulate networks of coupled non-linear oscillators with dependence on a number of factors (e.g., coupling strength, paradigmatic and empirical coupling topologies). We relate oscillators to vertices of a functional network and derive its edges with two widely used linear and non-linear estimators for the strength of interactions (maximum-lag cross correlation^75^ and mutual information^76^) between time series of the oscillators’ dynamics. We then examine how the aforementioned aspects of functional and structural networks coincide. We find that particularly local aspects of functional networks match ground truth to a high degree, while global aspects deviate.

## Results

### Simulation of complex dynamical systems

We simulate systems of *V* non-linear oscillators coupled onto networks with paradigmatic topologies (random, scale-free, and small-world; “Methods”). The equation of motion reads

1 $$\begin{aligned} \dot{{\mathbf {x}}}_i(t) = {\mathbf {f}}_i\left( {\mathbf {x}}_i(t)\right) + \varepsilon \sum _{j\ne i} ^V {\mathscr{A}} _{ij} {\mathbf {g}}\left( {\mathbf {x}}_i(t), {\mathbf {x}}_j(t)\right) , \end{aligned}$$

where ${\mathbf {x}}_i(t)$ denotes the state vector of the system’s *i*-th elementary unit. The function ${\mathbf {f}}_i\left( {\mathbf {x}}_i(t)\right)$ represents the dynamics of a Rössler oscillator, $\varepsilon$ denotes the global coupling strength, and ${\mathbf {g}}\left( {\mathbf {x}}_i(t), {\mathbf {x}}_j(t)\right)$ is the coupling function.

The networks are undirected, binary, and connected, and we refer to them as structural networks (${\mathscr{G}}^{{\text{s}}}$). They are represented by adjacency matrices ${\mathscr{A}} \in \{0,1\}^{V\times V}$, where each vertex *i* is associated with an oscillator. An edge between vertices *i* and *j* exists if and only if the corresponding oscillators are coupled. These are represented by entries ${\mathscr{A}} _{ij}=1$ and uncoupled oscillators by ${\mathscr{A}} _{ij}=0$. Note, that for $\varepsilon =0$ all oscillators are practically uncoupled even if structural networks would indicate a coupling according to ${\mathscr{A}} _{ij}=1$. We exclude self-loops with ${\mathscr{A}} _{ii} = 0\,\forall i$.

In order to prevent synchronization for weakly coupled and uncoupled oscillators, we draw natural frequencies randomly from ${\mathscr{N}}(1, \Delta \omega )$. For 20 realizations of each of the paradigmatic topologies, we generate time series of observables for various global coupling strengths $\varepsilon$ and various frequency inhomogeneities $\Delta \omega$. In the following, we set $V=50$ if not mentioned otherwise.

### Deriving functional networks

From theses time series, we derive functional networks ${\mathscr{G}}^{{\text{f}}}$ represented by weight matrices ${\mathscr{W}} \in {\mathbb {R}}_+^{V \times V}$, where each vertex corresponds to an oscillator and an edge represents the strength of interactions between pairs of oscillators. Simulating typical investigations of empirical data, we estimate the strength of interaction between pairs of oscillators (*i*, *j*) employing commonly used time-series-analysis techniques. Maximum-lag cross correlation $\sigma$^75^ is a linear estimator for synchronization and mutual information $\mu$^76^ also quantifies non-linear dependencies (“Methods”). Both these estimators are known to reliably assess the coupling strength from time-series data^77^. Both estimators are confined to the interval [0, 1], where 0 indicates no coupling. For $\sigma =1$ the two oscillators are lag-synchronized, while $\mu =1$ indicates the theoretical limit of information about an oscillator’s dynamics that one gains by observing the other. This maximum amount of information is achieved for identical time series with uniformly distributed amplitudes. By assigning values of $\sigma$ (or $\mu$) to the elements of the weight matrix ${\mathscr{W}}$, we derive a fully connected, undirected, and weighted functional network. In the following, functional networks derived with maximum-lag cross correlation are denoted by ${\mathscr{G}}^{{\text{f}}} _\sigma$ and those derived with mutual information by ${\mathscr{G}}^{{\text{f}}} _\mu$. An element ${\mathscr{W}} _{ij}$ then represents an edge weight in ${\mathscr{G}}^{{\text{f}}}$ and corresponds to an estimate of the strength of interaction—which in turn reflects coupling strength—between oscillators *i* and *j*. We set ${\mathscr{W}} _{ii}=0\,\forall i$ to exclude self-loops.

**Figure 1:**
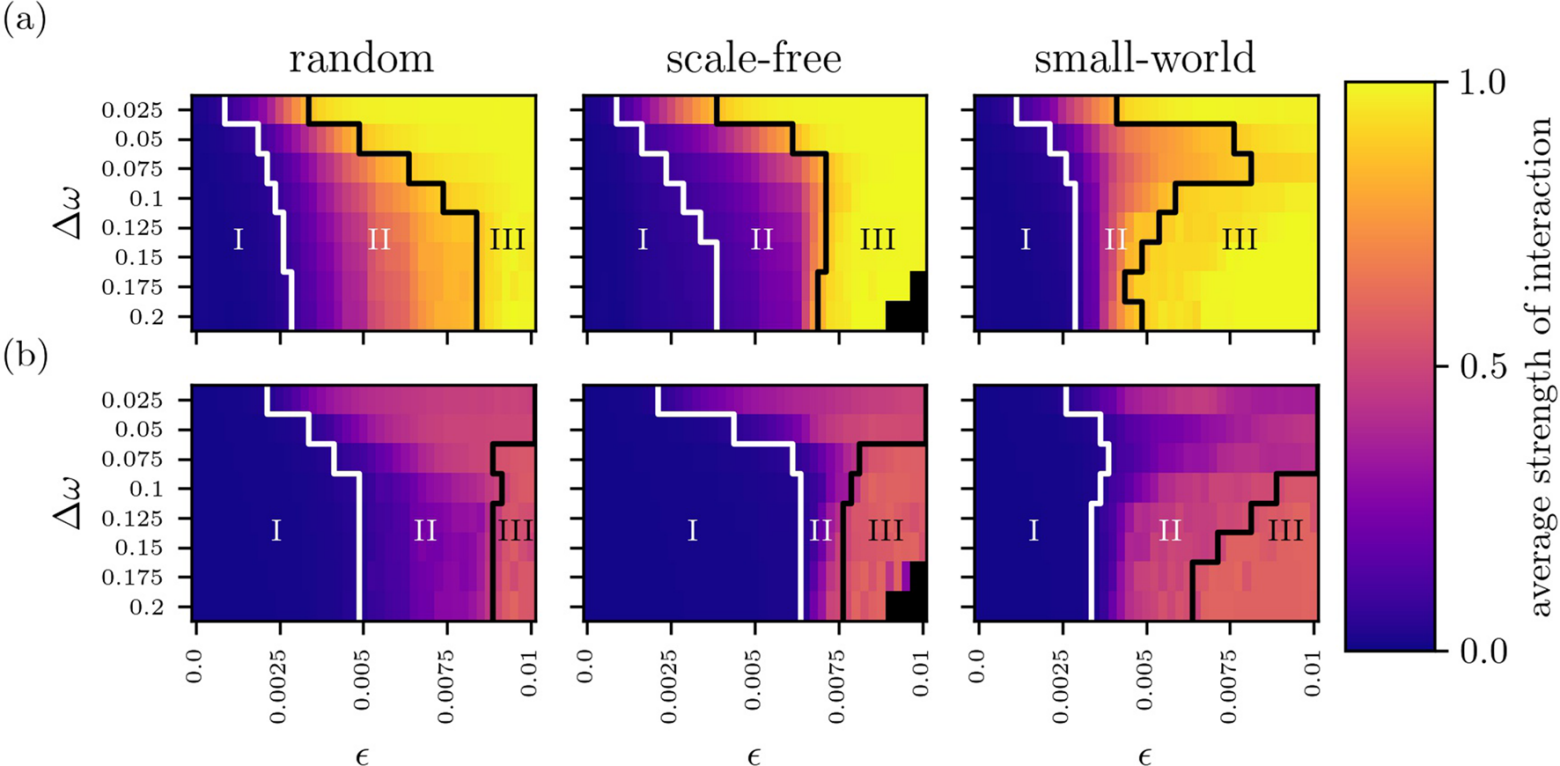
Average strength of interaction between oscillators (Eq. 2), “Methods” for various coupling topologies, coupling strengths $\varepsilon$, and frequency inhomogeneities $\Delta \omega$. Strength of interaction estimated with maximumlag cross correlation $\sigma$ (**a**) and with mutual information $\mu$ (**b**). Averages ($\left\langle {\overline{\sigma }} \right\rangle$ resp. $\left\langle {\overline{\mu }} \right\rangle$) derived from all non-redundant pairs of oscillators in a given network (size $V=50$) and from 20 realizations of network dynamics. For each realization of a coupling topology, initial conditions of oscillators were chosen randomly near the attractor. Coupling topologies are of random (left), scale-free (middle), or small-world type (right). Coupling strength $\varepsilon$ was varied from 0 to 0.01 in steps of 0.0002 and frequency inhomogeneity $\Delta \omega$ (width of the normal distribution of natural frequencies) from 0.025 to 0.2 in steps of 0.025. Outlined areas mark different regimes of coupling strengths (regime I: low, regime II: intermediate, and regime III: high). Black-colored pixels indicate parameter settings for which we only observed oscillation/amplitude death. Note, that the latter phenomenon is observed for high-degree nodes in networks with scale-free topologies. When increasing (decreasing) network size while keeping edge density constant, the borders between the regimes shift to lower (higher) coupling strengths and the distances between borders shrink (enlarge). Similar results can be expected for other types of oscillator dynamics^77^.

Coupling strength, coupling topology, and eigenfrequencies of oscillators are known to alter the collective dynamics of oscillator networks^1, 2, 78, 79^. Nevertheless, it is a priori not clear, how these control parameters influence estimates of the strength of interactions. More importantly, it can be expected that these (and other) control parameters also influence topological and spectral aspects of functional networks. Aspects that relate to the role of constituents in a network (e.g., involvement in shortest paths) are presumably influenced as well.

In Fig. 1, we report the influence of the aforementioned control parameters on the estimated strength of interactions (i.e., the edge weights). For all coupling topologies, strengths of interactions increase, on average, with increasing coupling strength. This dependence is more pronounced for small frequency inhomogeneities $\Delta \omega$. Within the chosen range of coupling strengths, an edge weight derived with maximum-cross correlation approaches its theoretical upper limit ($\sigma =1$) for large coupling strengths. In contrast, an edge weight derived with mutual information attains about two thirds of its upper limit ($\mu =1$). Despite this difference, we can subdivide the parameter space ($\varepsilon$, $\Delta \omega$) into three regimes of comparable ranges of edge weights.

- Regime I comprises low coupling strengths ($\varepsilon \lessapprox 0.003$ for $\sigma$ and $\varepsilon \lessapprox 0.005$ for $\mu$) and the full range of frequency inhomogeneities $\Delta \omega$ with low edge weights corresponding to weak strength of interactions ($0\le \eta \le 0.1\,\max _{\varepsilon ,\Delta \omega }(\eta )$; $\eta = \left\langle {\overline{\sigma }} \right\rangle$ resp. $\eta = \left\langle {\overline{\mu }} \right\rangle$). In this regime, edge weights within a network vary weakly. Increasing $\varepsilon$ only weakly increases edge weights.
- Regime II comprises intermediate coupling strengths ($0.003 \lessapprox \varepsilon \lessapprox 0.006$ for $\sigma$ and $0.005 \lessapprox \varepsilon \lessapprox 0.008$ for $\mu$) and the full range of frequency inhomogeneities $\Delta \omega$ with intermediate edge weights corresponding to intermediate strength of interactions ($0.1\,\max _{\varepsilon ,\Delta \omega }(\eta )<\eta \le 0.9\,\max _{\varepsilon ,\Delta \omega }(\eta )$). Here, the variability of edge weights is an order of magnitude larger compared to the other regimes. A small increase in $\varepsilon$ results, on average, in a strong increase of edge weights.
- Regime III comprises high coupling strengths ($\varepsilon \gtrapprox 0.006$ for $\sigma$ and $\varepsilon \gtrapprox 0.008$ for $\mu$) and the full range of frequency inhomogeneities for $\sigma$ resp. large frequency inhomogeneities ($\Delta \omega \gtrapprox 0.075$) for $\mu$. The regime corresponds to large and mostly similar edge weights ($0.9\,\max _{\varepsilon ,\Delta \omega }(\eta )<\eta \le \max _{\varepsilon ,\Delta \omega }(\eta )$). On average, increasing $\varepsilon$ only marginally increases edge weights.

In the following, we present our findings obtained from investigating all pairs of structural and functional networks from a given regime (see section Miscellaneous in “Methods” for the number of pairs per regime). We exclude only those pairs of ${\mathscr{G}}^{{\text{s}}}$ and ${\mathscr{G}}^{{\text{f}}}$ for which the corresponding oscillators exhibited oscillation/amplitude death^80^.

**Figure 2:**
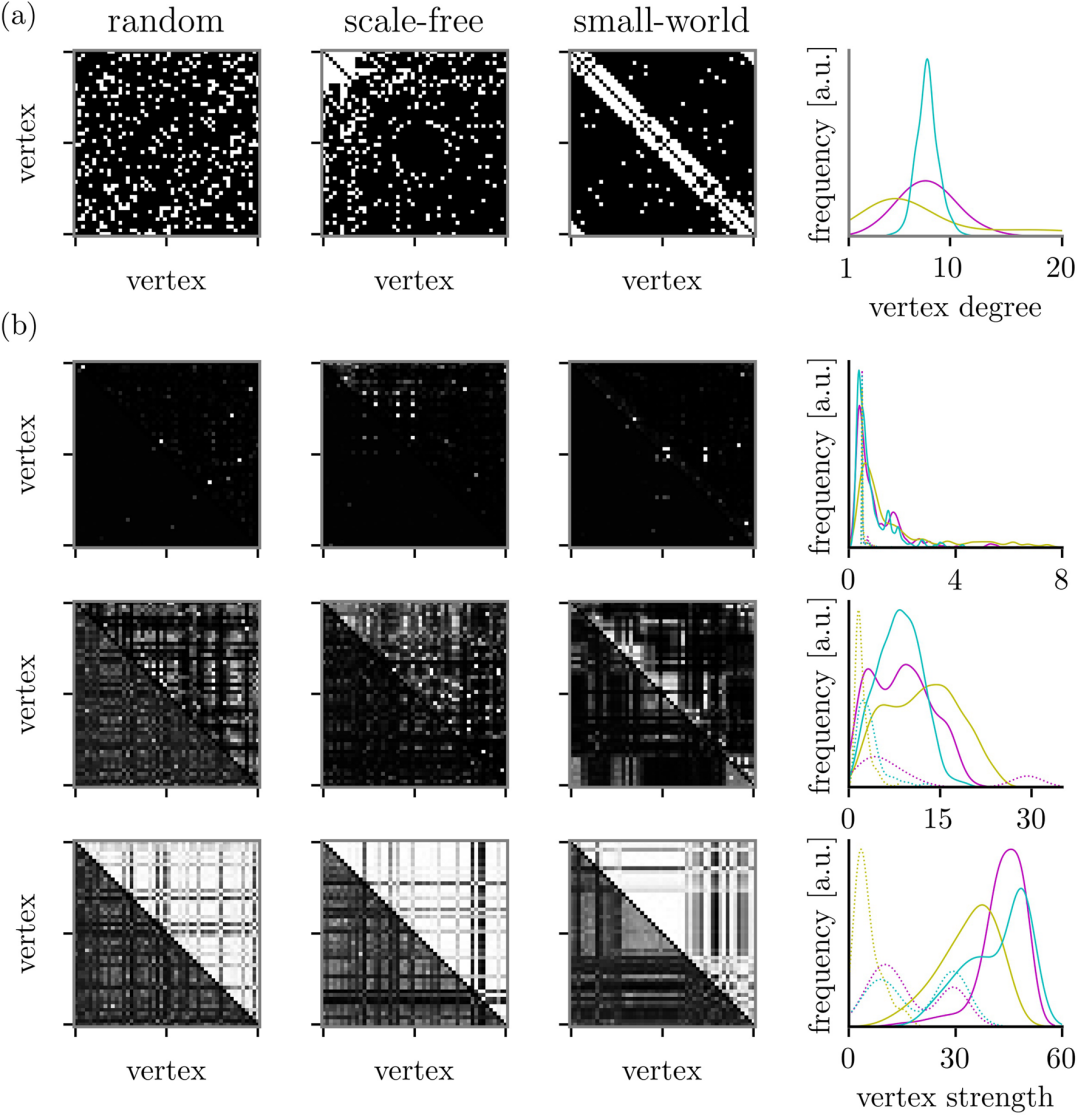
(**a**) Exemplary adjacency matrices of networks with paradigmatic coupling topologies. Matrix entries ${\mathscr{A}} _{ij} = 1$ are represented by white pixels and ${\mathscr{A}} _{ij} = 0$ by black pixels. (**b**) Weight matrices of corresponding functional networks with edge weights from the three regimes (top: regime I, middle: regime II, bottom: regime III). Coloring of matrix entries ranges from black (${\mathscr{W}} _{ij}=0$) to white (${\mathscr{W}} _{ij}=1$). The plots on the right side depict the associated degree resp. strength distributions (kernel density estimates). The degree of vertex *i* is defined as $\nu _i=\sum _{i\ne j}^V {\mathscr{A}} _{ij}$ and its strength as $s_i=\sum _{i\ne j}^V {\mathscr{W}} _{ij}$. Colors indicate coupling topologies (magenta: random, mustard: scale-free, and cyan: small-world). Weight matrices and strength distributions were derived with maximum lag-cross correlation (upper triangle of matrices; solid lines) and with mutual information (lower triangle of matrices; dashed lines).

To begin with, we show in Fig. 2 exemplary weight matrices corresponding to functional networks from the regimes together with the respective distributions of vertex strengths. Vertex strength is the sum of weights of edges connected to a vertex. For low coupling strengths, weight matrices are dominated by small edge weights with only rare entries of large weights, and correspondingly the distributions of vertex strengths center around small values. For intermediate coupling strengths, clusters of small numbers of vertices can be identified in the weight matrices, and the widths of distributions of vertex strengths are increased and centered around larger values. For high coupling strengths, entries of weight matrices are largely homogeneous, which leads to a seeming correspondence between adjacency and weight matrices. The widths of distributions of vertex strengths are further increased and center around even larger values.

### Evaluating concurrence between networks: from global to local scale

Comparing networks is often based on the use of distance metrics^81–86^. Available metrices, however, place strong assumptions on properties of networks (e.g., equal number of nodes or edge densities). In order to facilitate evaluating a concurrence between structural networks ${\mathscr{G}}^{{\text{s}}}$ and functional networks ${\mathscr{G}}^{{\text{f}}}$, we here employ measures that allow one to characterize different aspects of networks from the global to the local scale.

On the global scale, we make use of global clustering coefficient *C*, average shortest path length *L*, assortativity *A*, and synchronizability *S* (“Methods”). *C*, *L*, and *A* characterize topological and *S* spectral aspects of a network. Specifically, *C* measures the tendency of vertices to cluster together, and *L* measures the distance that, on average, has to be traversed to reach any vertex starting from any other vertex. Assortativity *A* quantifies the tendency of vertices to be connected to other vertices that share (dis-)similar properties^87, 88^ (here, vertex degree for ${\mathscr{G}}^{{\text{s}}}$ resp. vertex strength for ${\mathscr{G}}^{{\text{f}}}$), while *S* assesses the stability of a system’s synchronized state^89, 90^. We consider the relative difference between the respective global measures $\Delta M= (M_{{\text{f}}} - M_{{\text{s}}}) / \left|M_{{\text{s}}} \right|$ (*M* denotes a placeholder for the measures and $\left|\cdot \right|$ indicates the absolute value) from ${\mathscr{G}}^{{\text{s}}}$ and ${\mathscr{G}}^{{\text{f}}}$ and assume the respective network aspects to be similar if $\Delta M$ vanishes ($\left|\Delta M \right| < 5\%$).

On the local scale, we employ two centrality concepts to characterize the role of network constituents within the larger network (“Methods”). Betweenness centrality is a path-based concept and highlights a constituent as central if it acts as a bottleneck in a network. Eigenvector centrality is a degree-/strength-based concept and this centrality highlights a constituent as central if it is connected to other central constituents. Instead of considering differences between the centrality estimates of constituents from ${\mathscr{G}}^{{\text{s}}}$ and ${\mathscr{G}}^{{\text{f}}}$, we proceed differently. First, we estimate the correlation (Spearman’s $\rho$) between the ranked centrality values—for a given centrality concept—from ${\mathscr{G}}^{{\text{s}}}$ and from ${\mathscr{G}}^{{\text{f}}}$. To this end, we map constituents in ${\mathscr{G}}^{{\text{f}}}$ to constituents in ${\mathscr{G}}^{{\text{s}}}$. Since vertices in the networks are identical, the mapping is unique. For the calculation of the rank order of edges in ${\mathscr{G}}^{{\text{f}}}$, we omit the ones that have no counterpart in ${\mathscr{G}}^{{\text{s}}}$. Second, we estimate the length of the shortest path *d* between the most central (highest centrality value) constituents in ${\mathscr{G}}^{{\text{s}}}$ and ${\mathscr{G}}^{{\text{f}}}$. To this end, we calculate *d* as the number of edges along the shortest path in ${\mathscr{G}}^{{\text{s}}}$ between the most central vertex in ${\mathscr{G}}^{{\text{s}}}$ and the mapped one from ${\mathscr{G}}^{{\text{f}}}$ resp. as the number of vertices between the most central edge in ${\mathscr{G}}^{{\text{s}}}$ and the mapped one from ${\mathscr{G}}^{{\text{f}}}$. For the latter, we omit pairs of ${\mathscr{G}}^{{\text{s}}}$ and ${\mathscr{G}}^{{\text{f}}}$ for which the considered edge from ${\mathscr{G}}^{{\text{f}}}$ has no corresponding partner in ${\mathscr{G}}^{{\text{s}}}$. For $d=0$, most central constituents from ${\mathscr{G}}^{{\text{s}}}$ and ${\mathscr{G}}^{{\text{s}}}$ coincide, while for $d=1$ ($d=2$) most central constituents are nearest (next-nearest) neighbors in ${\mathscr{G}}^{{\text{s}}}$.

In the following sections, we report on the observed concurrences of network aspects between the structural and functional networks on different scales—from the global to the local one. We then evaluate the impact of uncertainties encountered in field applications on concurrences. Eventually, we extend our observations to an empirical network.

### Concurrences of global network aspects

**Figure 3:**
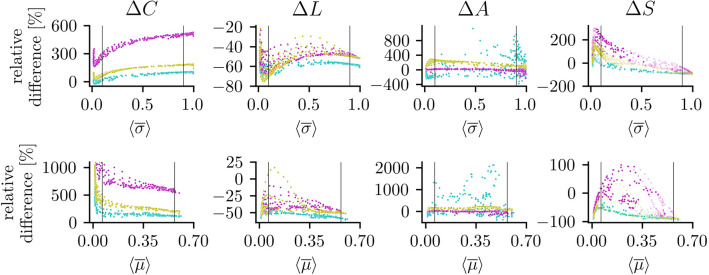
Relative difference $\Delta M$ of global network aspects depending on average strength of interactions. *M* is a placeholder for measures of global network aspects: global clustering coefficient *C*, average shortest path length *L*, assortativity *A*, and synchronizability *S*. Relative differences are averages derived from 20 realizations of network dynamics, and colors indicate types of coupling topology (magenta: random, mustard: scale-free, and cyan: small-world). Average strength of interaction estimated with maximum-lag cross correlation ($\left\langle {\overline{\sigma }} \right\rangle$; top) and mutual information ($\left\langle {\overline{\mu }} \right\rangle$; bottom; cf. Fig. 1). Vertical lines indicate borders between regimes I and II and between regimes II and III. For $\Delta S$, we additionally indicate with the transparency of dots the correlation between eigenvalue spectra of the structural and the functional networks’ Laplacian matrices: solid dots indicate a Pearson correlation coefficient of larger than 0.98 and faded dots illustrate a lower correlation coefficient.

As regards the topological aspects of the structural and functional networks, we observe the global clustering coefficients *C* of ${\mathscr{G}}^{{\text{f}}}$ to mostly exceed those of ${\mathscr{G}}^{{\text{s}}}$ (see Fig. 3; for reference, global network aspects of ${\mathscr{G}}^{{\text{s}}}$ are listed in Table 1a). This holds true for a wide range of average strength of interactions, independent of the employed estimator for the latter, and for all coupling topologies. The relative differences between the respective global clustering coefficients $\Delta C$ only vanishes for small-world coupling topologies with low coupling strengths (regime I) and when ${\mathscr{G}}^{{\text{f}}}$ is derived with $\sigma$, which indicates the respective structural and functional networks to share a similar tendency for vertices to cluster together. Likewise, the average shortest path length *L* is mostly shorter in ${\mathscr{G}}^{{\text{f}}}$, which can be attributed to the fact that functional networks are fully connected, by construction. Assortativity values of ${\mathscr{G}}^{{\text{f}}}$ based on random coupling topologies match the range of values of the corresponding structural networks ${\mathscr{G}}^{{\text{s}}}$ independent of strength of interactions and of estimator for the latter. In contrast, ${\mathscr{G}}^{{\text{f}}}$ based on scale-free coupling topologies tend to be more assortative than their, on average, disassortative structural counterparts, while the opposite holds true for small-world coupling topologies. Again, these relationships are independent of strength of interactions and of the estimator for the latter. We conjecture, that the reported similarities and dissimilarities between the topological aspects of structural and functional networks can be traced back—to quite a large extent—to properties of the weight distribution of ${\mathscr{G}}^{{\text{f}}}$ such as the width and central value (cf. Fig. 2).

**Table 1:** Global network aspects of structural networks (a) and relative frequency of vanishing relative differences of global network aspects for ${\mathscr{G}}^{{\text{f}}} _\sigma$ (b) and for ${\mathscr{G}}^{{\text{f}}} _\mu$ (c).

(a)	*C*			*L*			*A*			*S*		
Random	0.16 (0.13, 0.21)			2.06 (2.05, 2.08)			$-0.04~(-0.15, 0.08)$			7.21 (5.70, 21.98)		
Scale-free	0.34 (0.31, 0.45)			2.05 (1.97, 2.08)			$-0.13~(-0.28, -0.06)$			11.00 (9.27, 13.39)		
Small-world	0.48 (0.44, 0.55)			2.41 (2.32, 2.60)			$-0.02~(-0.15, 0.84)$			12.5 (10.6, 18.1)		

(b)	$\left|\Delta C \right| < 5\%$			$\left|\Delta L \right| < 5 \%$			$\left|\Delta A \right| < 5 \%$			$\left|\Delta S \right| < 5 \%$		
	I	II	III	I	II	III	I	II	III	I	II	III
Random	0	0	0	0.1	0	0	0.5	0.7	0.6	1.3	5.7	0.8
Scale-free	0.8	0	0	0	0	0	0	0	0	2.5	7.4	0
Small-world	16.9	1.1	0	0	0	0	1.9	1.5	1.2	9.3	2.0	0

(c)	$\left|\Delta C \right| < 5 \%$			$\left|\Delta L \right| < 5 \%$			$\left|\Delta A \right| < 5 \%$			$\left|\Delta S \right| < 5\%$		
	I	II	III	I	II	III	I	II	III	I	II	III
Random	0	0	0	0.3	1.2	0	0.8	0.8	0.8	2.9	3.7	0
Scale-free	0	0	0	0.4	1.4	0	0	0	0	0.3	1.9	0
Small-world	0	0	0	0.1	0	0	1.7	1.2	0	0.1	0.2	0

(a) Median and range (in brackets) of measures of global network aspects (global clustering coefficient *C*, average shortest path length *L*, assortativity *A*, and synchronizability *S*) from 20 realizations of paradigmatic coupling topologies (small-world, random, and scale-free). (b) and (c) Percentage of pairs of a structural and a functional network for which absolute values of relative difference vanish (i.e., $\left|\Delta M \right| < 5\%$; *M* denotes a placeholder for measures of global network aspects). The pairs are broken down according to the regimes of low (I), of intermediate (II), and of high coupling strengths (III).

**Figure 4:**
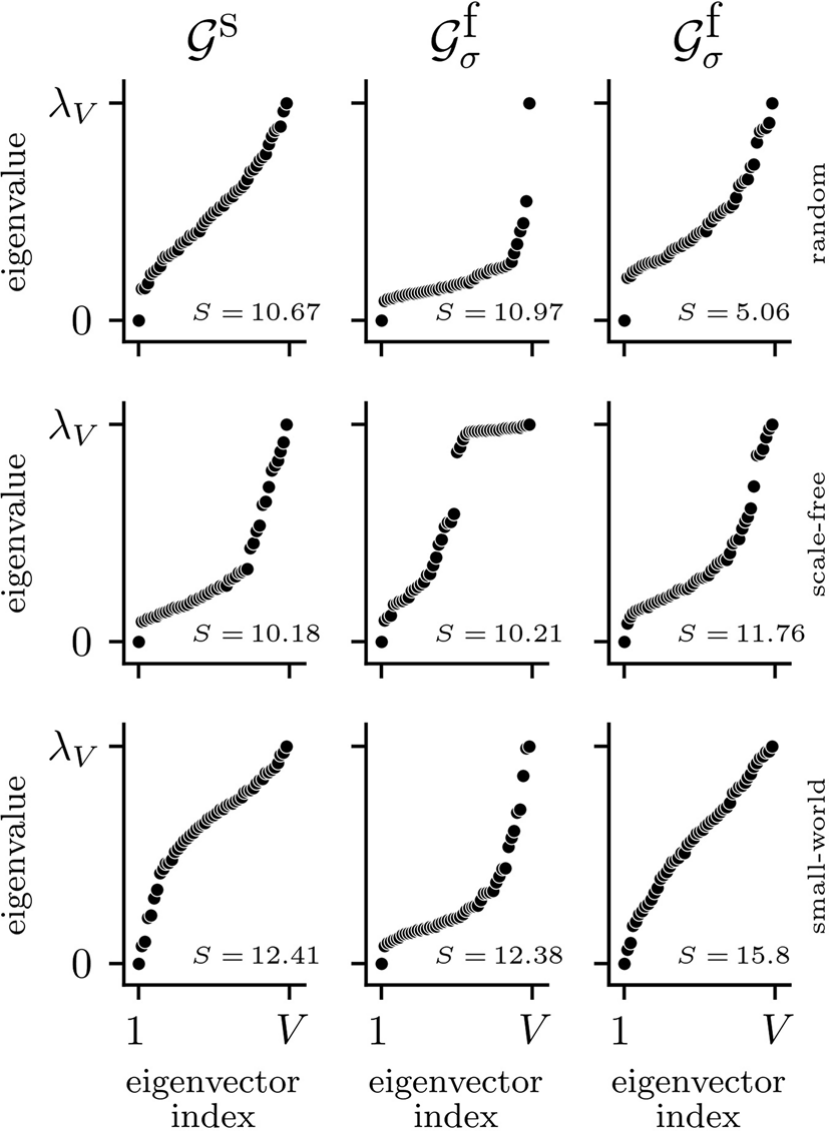
Exemplary eigenvalue spectra of Laplacian matrices of structural networks (${\mathscr{G}}^{{\text{s}}}$; left column) and of corresponding functional networks (${\mathscr{G}}^{{\text{f}}} _\sigma$; middle and right columns). Middle column: ${\mathscr{G}}^{{\text{s}}}$ and ${\mathscr{G}}^{{\text{f}}} _\sigma$ have comparable synchronizability *S* (relative difference between eigenratios $\Delta S<5\%$). Right column: ${\mathscr{G}}^{{\text{s}}}$ and ${\mathscr{G}}^{{\text{f}}} _\sigma$ have comparable eigenvalue spectra (Pearson correlation coefficient $> 0.98$). Similar findings were achieved with mutual information.

As regards the spectral aspect of the structural and functional networks (see Fig. 3, Table 1), we find synchronizability *S* of ${\mathscr{G}}^{{\text{s}}}$ to be smallest for random coupling topologies and to be higher for scale-free and small-world topologies^91, 92^. For ${\mathscr{G}}^{{\text{f}}}$, *S* is strongly influenced by coupling topology as well as strengths of interaction^89^. For ${\mathscr{G}}^{{\text{f}}} _\sigma$, $\Delta S$ vanishes for small-world coupling topologies with intermediate coupling strengths (regime II). For ${\mathscr{G}}^{{\text{f}}} _\mu$, $\Delta S$ vanishes for some cases of random topologies with low and intermediate coupling strengths (regimes I and II). While we also find vanishing $\Delta S$ for other coupling topologies, in these cases the eigenvalue spectra of the Laplacian matrices of ${\mathscr{G}}^{{\text{s}}}$ and ${\mathscr{G}}^{{\text{f}}}$ differ strongly (see Fig. 4), which does not allow for a reasonable comparison of the synchronizability values. In contrast, we often observe eigenvalue spectra of ${\mathscr{G}}^{{\text{s}}}$ and ${\mathscr{G}}^{{\text{f}}}$ to be similar but with non-vanishing $\Delta S$ for the regimes of low and intermediate coupling strengths. Independent of the estimator used to derive ${\mathscr{G}}^{{\text{f}}}$ and of coupling topology, *S* of ${\mathscr{G}}^{{\text{f}}}$ converges to 1 for high coupling strengths (regime III), which is a direct consequence of properties of the weight distribution of ${\mathscr{G}}^{{\text{f}}}$: for $\left\langle {\overline{\mu }} \right\rangle \rightarrow 1$ resp. $\left\langle {\overline{\mu }} \right\rangle \rightarrow 0.65$, all edge weights are identical (cf. Fig. 2) and the eigenvalue spectra of the functional networks’ Laplacian matrices are degenerate.

Overall, our findings indicate structural and functional networks to clearly differ in their global aspects investigated here. The observed coincidence of the global clustering coefficients seen in about one sixth of structural networks with small-world coupling topology and their corresponding functional networks when coupling strengths are low (see Table 1b and c) may be attributed to the choice of the rewiring probability (here $p_{{\text{sw}}} = 0.1$). Thus, this coincidence should not be taken as an indicator for an equal tendency of vertices to cluster together. We note, that we obtained similar findings for networks composed of $V=25$ or $V=100$ oscillators.

### Concurrences of local network aspects

**Figure 5:**
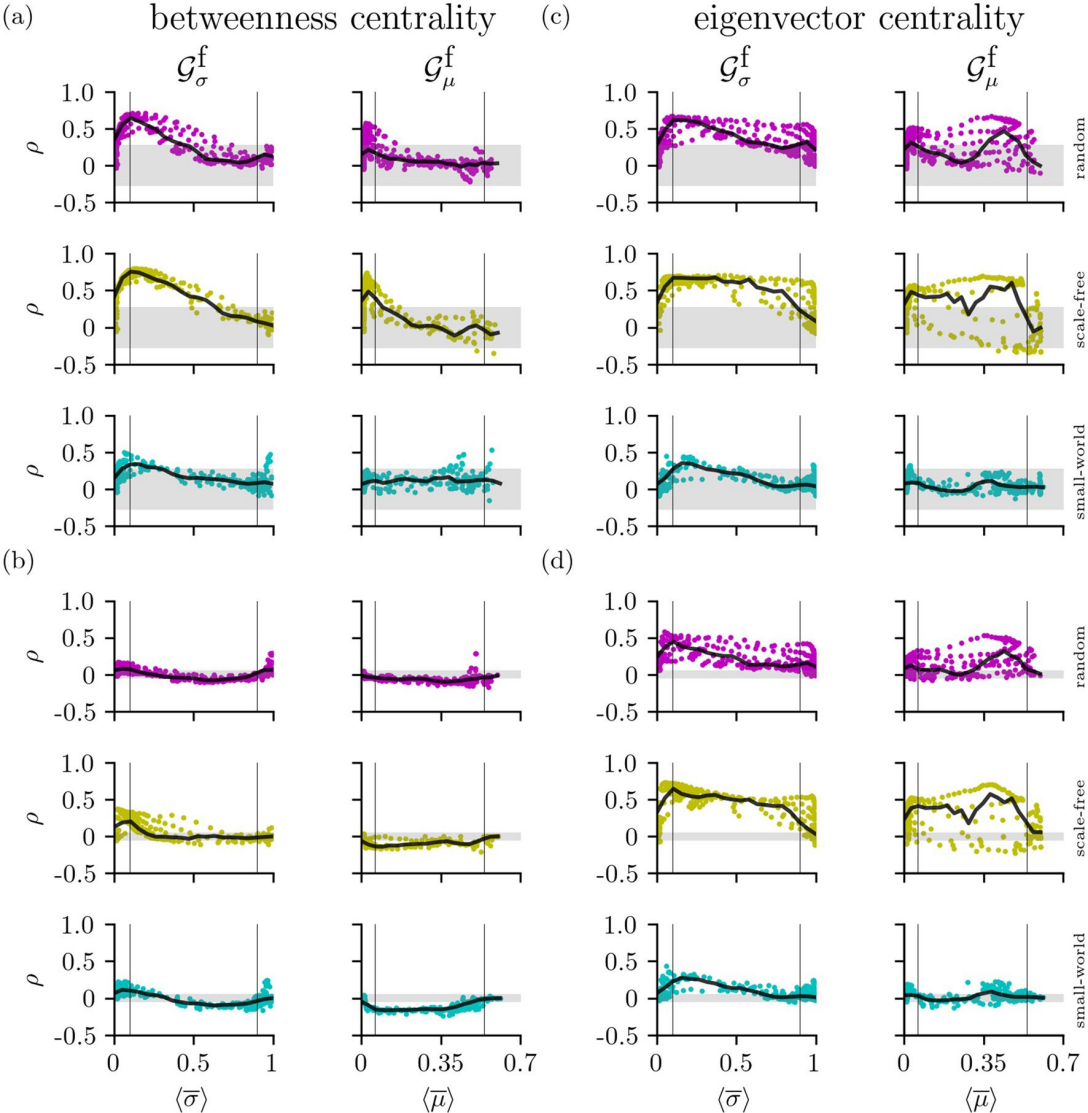
Spearman’s rank correlation coefficient $\rho$ of vertex (**a**,**c**) resp. edge (**b**,**d**) centrality values between structural and functional networks depending on average strengths of interaction (derived with either maximum-lag cross correlation $\sigma$ or mutual information $\mu$). Rankings are based on either betweenness centrality (**a**,**b**) or eigenvector centrality (**c**,**d**). Vertical lines indicate borders between regimes I and II and between regimes II and III, and grey-shaded areas indicate critical values of $\rho$ for a significance level of $\alpha =0.05$ (calculated from Student’s *t*-distribution^93^). Each data point is an average over 20 realizations with identical control parameter settings and randomized initial conditions near the attractors of the systems. Semi-transparent black lines are moving averages of $\rho$ derived from an equidistant binning of the range of average strength of interactions (20 bins; lines are for eye-guidance only).

On the next smaller network scale, we investigate whether the ranking of network constituents in ${\mathscr{G}}^{{\text{f}}}$ is congruent with the one in ${\mathscr{G}}^{{\text{s}}}$ (see Fig. 5). For the networks investigated here, we do not observe congruent rankings of the respective constituents.

Nevertheless, with the path-based betweenness centrality (Fig. 5a), we find (statistically significant) linear relationships ($0.3<\rho < 0.8$) between rankings of the networks’ vertices for low to intermediate coupling strengths (${\mathscr{G}}^{{\text{f}}} _\sigma$: border between regimes I and II; ${\mathscr{G}}^{{\text{f}}} _\mu$: most of regime I; see also Table 2). These relationships are most pronounced and seen more often for networks with random and scale-free coupling topologies. As regards the networks’ edges (Fig. 5b), there are at best weak associations between rankings independent of coupling strength and topology.

With the strength-based eigenvector centrality (Fig. 5c), relationships are in general less pronounced than with betweenness centrality but extend over most of the range of coupling strengths for networks with random and scale-free coupling topologies. Relationships seen for the networks’ edges compare to the ones seen for the networks’ vertices (Fig. 5d).

We conjecture that the observed relationships between the rankings of the networks’ constituents can be traced back, at least to some extent, to the width of the distributions of vertex degrees being more narrow for small-world coupling topologies compared to the other ones (cf. Fig. 2; note, that the mean degree is identical for all topologies). A more narrow degree distribution results in a more comparable dynamics among oscillators even for lower coupling strengths. This renders a distinction between constituents in ${\mathscr{G}}^{{\text{f}}}$ (in the sense of centrality) more difficult, thus leading to an ambiguous ranking of vertex and edge centralities in ${\mathscr{G}}^{{\text{f}}}$.

The weak association seen for the path-based edge betweenness centralities from ${\mathscr{G}}^{{\text{s}}}$ and ${\mathscr{G}}^{{\text{f}}}$ indicates that—given our scheme of comparison—edges forming shortest paths in the fully connected ${\mathscr{G}}^{{\text{f}}}$ may not have counterparts in the sparse ${\mathscr{G}}^{{\text{s}}}$. These surplus edges, nonetheless, modify the collection of shortest paths in ${\mathscr{G}}^{{\text{f}}}$ and, consequentially, impact on the rankings of the other edges. This impact is less pronounced in case of the strength-based edge eigenvector centrality.

We note, that the observed relationships depend on the size of the networks being more pronounced for smaller networks ($V=25$) and slightly less pronounced for larger networks ($V=100$).

**T﻿able 2:** Percentage of pairs of structural and functional networks for which the rank orders of vertex resp. edge centrality values are positively correlated (Spearman’s $\rho$; $p<0.05$).

${\mathscr{G}}^{{\text{f}}} _\sigma$	Betweenness centrality					
	Vertex			Edge		
	I	II	III	I	II	III
Random	**73.6**	44.5	0.1	58.7	19.4	10.4
Scale-free	**86.4**	**80.9**	0.2	**75.7**	48.9	0.8
Small-world	31.3	26.3	0.0	**67.3**	23.8	0.1

${\mathscr{G}}^{{\text{f}}} _\mu$	Betweenness centrality					
	Vertex			Edge		
	I	II	III	I	II	III
Random	44.1	6.3	0.0	19.6	4.5	0.0
Scale-free	**77.7**	9.9	0.0	9.1	2.5	0.0
Small-world	8.6	9.6	0.0	16.6	1.6	0.0

${\mathscr{G}}^{{\text{f}}} _\sigma$	Eigenvector centrality					
	Vertex			Edge		
	I	II	III	I	II	III
Random	53.6	**77.5**	53.2	**78.5**	**82.3**	**71.7**
Scale-free	**81.6**	**95.2**	42.2	**86.9**	**98.4**	64.8
Small-world	22.2	41.1	16.7	52.5	60.3	46.4

${\mathscr{G}}^{{\text{f}}} _\mu$	Eigenvector centrality					
	Vertex			Edge		
	I	II	III	I	II	III
Random	52.4	58.1	17.6	61.6	**73.1**	52.4
Scale-free	**72.0**	**74.1**	13.7	**75.2**	**84.3**	47.4
Small-world	22.3	33.4	3.2	49.0	48.1	39.4

Rankings are based on either betweenness centrality or eigenvector centrality. Upper part: functional networks ${\mathscr{G}}^{{\text{f}}} _\sigma$ derived with maximum-lag cross correlation; lower part: ${\mathscr{G}}^{{\text{f}}} _\mu$ derived with mutual information . The pairs are broken down according to the regimes of low (I), of intermediate (II), and of high coupling strengths (III). We highlight cases in bold for which percentages exceed $66.6\%$.

### Concurrences of most central constituents

**Figure 6:**
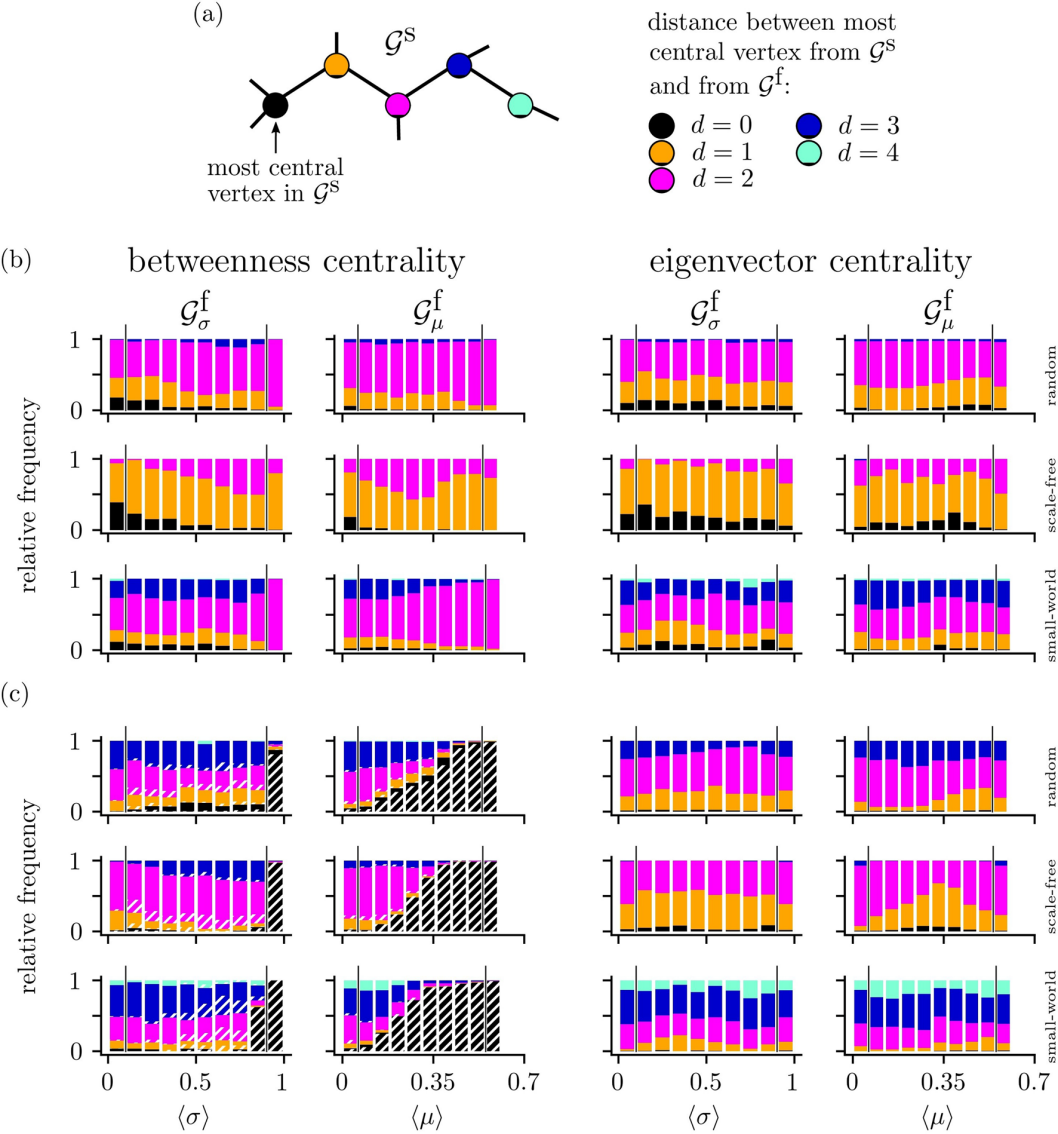
(**a**) Schematic of estimating the distance *d* between the most central constituents—here vertices—from a structural network ${\mathscr{G}}^{{\text{s}}}$ and from a functional network ${\mathscr{G}}^{{\text{f}}}$. The coloring of a vertex indicates the distance between the most central vertex in ${\mathscr{G}}^{{\text{s}}}$ and the most central one from a given realization of ${\mathscr{G}}^{{\text{f}}}$ mapped onto ${\mathscr{G}}^{{\text{s}}}$. Coinciding most central constituents ($d=0$) are shown in black, most central constituents being nearest neighbors ($d=1$) in orange or being next-nearest neighbors ($d=2$) in pink. More distant central constituents are colored dark blue ($d=3$) or lightblue ($d\ge 4$). (**b**,**c**) Stacked histograms of the relative frequency of distances between the most central vertex (**b**)/edge (**c**) from ${\mathscr{G}}^{{\text{f}}}$ and the most central constituent from the corresponding ${\mathscr{G}}^{{\text{s}}}$ depending on average strength of interactions. Coloring as in (**a**). Hatched bars indicate functional networks ${\mathscr{G}}^{{\text{f}}}$, for which a most central constituent could not be identified unambiguously. Black vertical lines between the bars indicate borders between regimes I and II and between regimes II and III.

Eventually, we investigate whether the most central (i.e., rank 1) constituent of a functional network ${\mathscr{G}}^{{\text{f}}}$ coincides with the most central one of the corresponding structural network ${\mathscr{G}}^{{\text{s}}}$. Interestingly, for network vertices (see Fig. 6a), we find coincidences with both vertex centrality measures for all coupling topologies and for all but predominantly for low strengths of interactions (with both estimators for the latter). Coincidences are most often (up to $39\%$) encountered for scale-free coupling topologies, followed by random and small-world topologies, and even for these cases, coincidences are encountered more often than to be expected by chance ($2\%$). Of note, if most central vertices do not coincide, we predominantly find them to be nearest or next-nearest neighbors in ${\mathscr{G}}^{{\text{s}}}$ (chance levels for nearest neighbors: $16\%$ for all topologies; chance levels for for next-nearest neighbors: $61\%$ for random topologies, $63\%$ for scale-free topologies, and $34\%$ for small-world topologies).

As regards network edges (see Fig. 6b), we observe that the most central edge in ${\mathscr{G}}^{{\text{f}}}$ has no corresponding partner in ${\mathscr{G}}^{{\text{s}}}$ in, on average, $40\%$ of pairs of ${\mathscr{G}}^{{\text{s}}}$ and ${\mathscr{G}}^{{\text{f}}}$. This rate is smallest for ${\mathscr{G}}^{{\text{f}}} _\sigma$ based on scale-free coupling topologies and when edge centrality is estimated with betweenness centrality ($18\%$). It is highest for ${\mathscr{G}}^{{\text{f}}} _\mu$ based on random coupling topologies with and when edge centrality is estimated with eigenvector centrality ($58\%$). In the remaining cases, the frequency of coincidences is strongly reduced, but nevertheless still exceeds chance level ($0.5\%$). Again, coincidences are seen more often for scale-free coupling topologies, followed by random and small-world topologies. On average, more than half of the non-coinciding most central edges are nearest or next-nearest neighbors in ${\mathscr{G}}^{{\text{s}}}$.

A smaller size of the networks ($V=25$) results in a comparable number of coincidences for both vertices and edges, while we observed a reduced number of coincidences for larger networks ($V=100$), that still exceed chance levels.

### Impact of noise

With an eye on field applications and given the various degrees of observed similarities between aspects of structural and functional networks, we evaluate how uncertainties in deriving the functional networks impact on these similarities. To this end, we simulate uncertainties of increasing severity $q$ by adding Gaussian distributed white noise to the entries of the weight matrices. Noise amplitudes were drawn from the normal distribution ${\mathscr{N}}(0, \eta q)$, where $\eta$ denotes the average strength of interaction ($\eta = \left\langle {\overline{\sigma }} \right\rangle$ resp. $\eta = \left\langle {\overline{\mu }} \right\rangle$; cf. Fig. 1; $q\in \left\{ 0.01,0.02, 0.05, 0.1, 0.2, 0.5, 1.\right\}$). In order to facilitate a comparison between noise-free and noisy weight matrices, we limited noisy weights to the range of weights observed for noise-free cases. In the following, we restrict investigations to the regimes of low and intermediate coupling strengths (regimes I and II), i.e., the regimes for which we observed highest similarities between aspects of ${\mathscr{G}}^{{\text{s}}}$ and ${\mathscr{G}}^{{\text{f}}}$.

For both the topological and spectral network aspects, we generally find similarities seen for the noise-free cases to worsen with increasing the severity of uncertainty. Dissimilar cases remained dissimilar and uncertainties did not positively affect these cases. As regards assortativity *A*, for functional networks based on small-world or scale-free coupling topologies *A* takes on values seen for random coupling topologies when increasing the severity of uncertainty.

We obtained comparable findings for the relationships between rankings of centralities of network constituents. Increasing the severity of uncertainty above one fifth of the average strengths of interactions obfuscates previous statistically significant relationships in the majority of cases.

Interestingly, on the level of most central network vertices but not edges, we find coincidences to be largely unaffected by the same range of uncertainties. This holds true particularly for strengths of interactions from regime I and if estimated with maximum-lag cross correlation. Coincidences remained in a similar range of uncertainties, however, for random and scale-free coupling topologies only if strengths of interactions were estimated with mutual information.

### Empirical network

**Figure 7:**
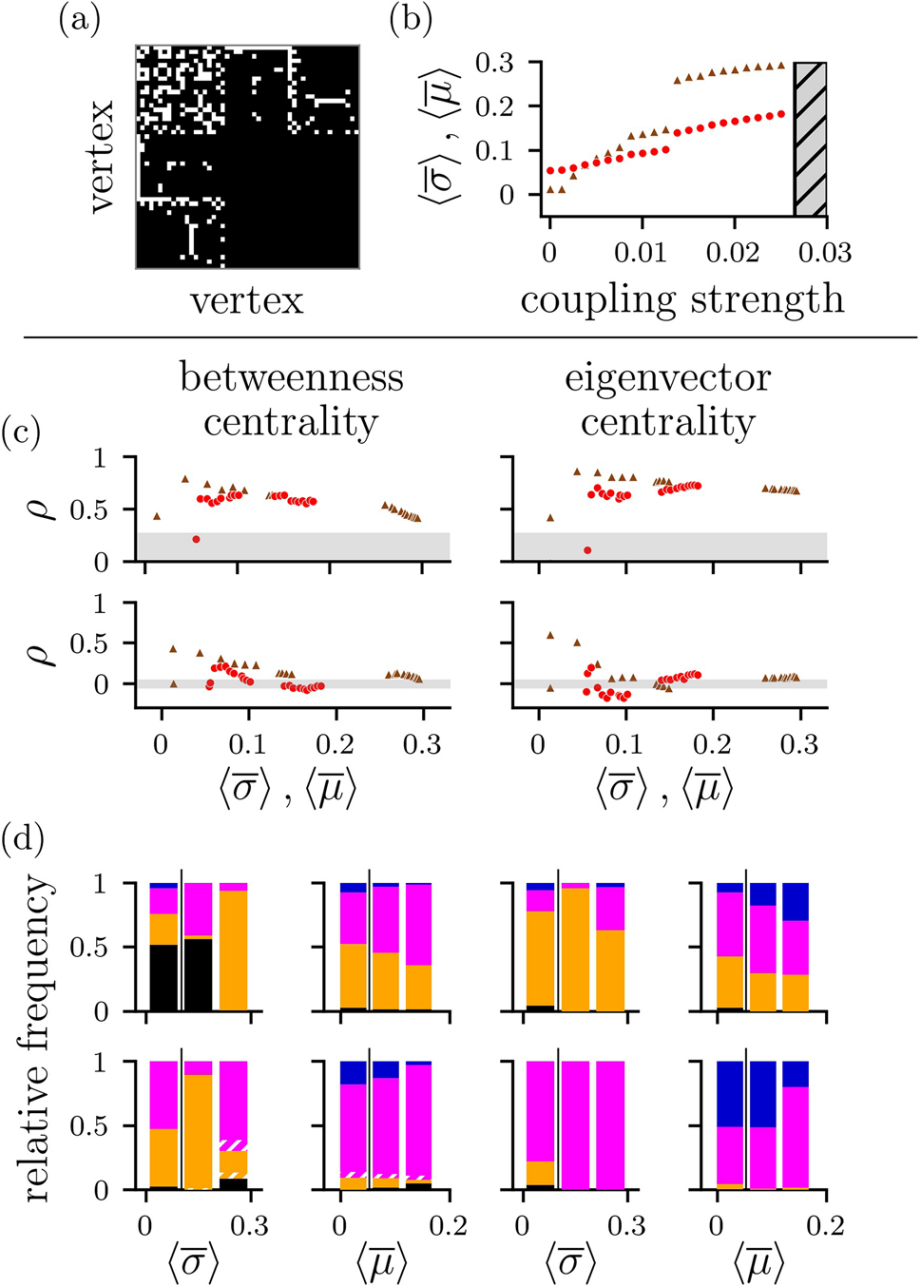
Concurrences of local aspects between an empirical structural network ${\mathscr{G}}^{{\text{s}}} _{{\text{emp}}}$ (cell network of P. pacificus^94^) and functional networks ${\mathscr{G}}^{{\text{f}}}$ derived from time series of oscillators coupled onto ${\mathscr{G}}^{{\text{s}}} _{{\text{emp}}}$. (**a**) Adjacency matrix of ${\mathscr{G}}^{{\text{s}}} _{{\text{emp}}}$. (**b**) Average strength of interaction between oscillators for various coupling strengths $\varepsilon$. Strength of interactions estimated with maximum-lag cross correlation $\sigma$ (brown triangles) and with mutual information $\mu$ (red dots). Each data point is an average over 20 realizations with identical control parameter settings and randomized initial conditions. Coupling strength was varied from 0 to 0.05 in steps of 0.00125 (cf. Fig. 1; frequency inhomogeneity $\Delta \omega =0.125$). Hatched area indicates range of coupling strengths for which oscillation/amplitude death occurred. (**c**) Spearman’s rank correlation coefficient $\rho$ of vertex (top) and edge (bottom) centrality values between ${\mathscr{G}}^{{\text{s}}} _{{\text{emp}}}$ and ${\mathscr{G}}^{{\text{f}}}$ depending on average strength of interaction ($\sigma$: brown triangles; $\mu$: red dots). Otherwise, same as Fig. 5. (**d**) Relative frequency of distances between the most central vertex (top) and edge (bottom) from ${\mathscr{G}}^{{\text{f}}}$ and the most central constituent from ${\mathscr{G}}^{{\text{s}}} _{{\text{emp}}}$ depending on average strength of interaction. Otherwise, same as Fig. 6b,c.

We use a small cell network of a P. pacificus nematode^94^ as a structural network ${\mathscr{G}}^{{\text{s}}} _{{\text{emp}}}$ and as before derive functional networks ${\mathscr{G}}^{{\text{f}}}$ from time series of oscillators coupled onto ${\mathscr{G}}^{{\text{s}}} _{{\text{emp}}}$. The network comprises $V=50$ vertices and $E=141$ edges (average vertex degree $\langle \nu \rangle =5.64$) with a topology that deviates from the other employed topologies (cf. Figs. 2a, 7a): it is neither random nor entirely regular and has a large fraction of vertices with small vertex degree as well as rare vertices with a high vertex degree without exhibiting a scale-free degree distribution.

Notably, we observe oscillation/amplitude death for coupling strengths $\varepsilon \ge 0.0265$, which we assume to be caused by the specific topology (in combination with a preset frequency inhomogeneity of $\Delta \omega =0.125$). This limits the maximum of the estimated strength of interactions to ${{\text{max}}}(\left\langle \sigma \right\rangle )=0.29$ resp. ${{\text{max}}}(\left\langle \mu \right\rangle )=0.18$ (see Fig. 7b) and, consequently, we restrict our following investigations to the regimes of weak and intermediate coupling strengths (regimes I and II; “Methods”).

On the global scale, we again find topological and spectral aspects of functional networks to deviate from ground truth. The global clustering coefficient *C* of ${\mathscr{G}}^{{\text{s}}} _{{\text{emp}}}$ ($C=0.33$) is exceeded by the ones of ${\mathscr{G}}^{{\text{f}}} _\sigma$ (on average, $\Delta C = 92\%$) and of ${\mathscr{G}}^{{\text{f}}} _\mu$ (on average, $\Delta C = 58\%$). The average shortest path length *L* of ${\mathscr{G}}^{{\text{s}}} _{{\text{emp}}}$ ($L=2.47$) either exceeds the one from ${\mathscr{G}}^{{\text{f}}} _\sigma$ (on average, $\Delta L = -70\%$) or falls below the one from ${\mathscr{G}}^{{\text{f}}} _\mu$ (on average, $\Delta L = 144\%$). Regarding assortativity *A*, ${\mathscr{G}}^{{\text{s}}} _{{\text{emp}}}$ is assortative ($A=0.19$; it exceeds the range of values $[-0.21, 0.16]$ expected for random networks with the same number of vertices and edges), while assortativity values of ${\mathscr{G}}^{{\text{f}}} _\sigma$ and of ${\mathscr{G}}^{{\text{f}}} _\mu$ are smaller (on average, $\Delta A =-48\%$ resp. $\Delta A= -67\%$). The synchronizability *S* of ${\mathscr{G}}^{{\text{s}}} _{{\text{emp}}}$ is unexpectedly large ($S=48.9$; see Table 1 for comparison) and is generally larger than the ones of ${\mathscr{G}}^{{\text{f}}} _\sigma$ and of ${\mathscr{G}}^{{\text{f}}} _\mu$ (on average, $\Delta S =-60.9\%$ resp. $\Delta S=-89.7 \%$). We conjecture that this particularly large synchronizability is the reason, why we encounter oscillation/amplitude death for larger coupling strengths.

On the local scale, we find significant linear relationships of betweenness-centrality- and eigenvector-centrality-based rankings ($0.3<\rho <0.92$) of vertices between ${\mathscr{G}}^{{\text{s}}} _{{\text{emp}}}$ and ${\mathscr{G}}^{{\text{f}}}$ for both low and intermediate coupling strengths (see Fig. 7c,d). For edges, there are again at best weak associations between these rankings. Overall, the relationships are largely independent of the estimators used to derive functional networks as well as of the centrality measure used to rank constituents, and they generally compare to the ones seen for structural networks of scale-free type.

The most central constituents of ${\mathscr{G}}^{{\text{f}}}$ coincide with the most central ones of ${\mathscr{G}}^{{\text{s}}} _{{\text{emp}}}$ either more frequently ($\approx 50\%$ of pairs ${\mathscr{G}}^{{\text{s}}} _{{\text{emp}}}$ and ${\mathscr{G}}^{{\text{f}}} _\sigma$ for low coupling strengths when vertices are ranked with betweenness centrality; see Fig. 7d) or less frequently (other cases) than observed for the paradigmatic coupling topologies (cf. Fig. 6).

Non-coinciding most central vertices and edges of ${\mathscr{G}}^{{\text{f}}} _\sigma$ resp. ${\mathscr{G}}^{{\text{f}}} _\mu$ are also predominantly nearest or next-nearest neighbors to the most central ones from ${\mathscr{G}}^{{\text{s}}} _{{\text{emp}}}$.

## Discussion

Knowledge about the organization of a structural network representing a complex dynamical system is critically important to gain deeper understanding of the system’s dynamics, thereby providing improved means for prediction and control. In many natural and man-made systems, however, access to this organization is usually limited due to various reason. In these cases, an alternative approach is to resort to analyzing interactions between system units to draw conclusions about the organization of a structural network. Despite some progress in this active field of research, there are still problems that evade from a satisfactory solution and it remains unclear if and, particularly, to what extent this organization can be revealed.

To tackle this issue, we simulated oscillator networks with preset (paradigmatic and empirical) coupling topologies—i.e., structural networks—and derived functional networks from interactions estimated from time series of the oscillators’ dynamics under idealized conditions. We then evaluated to which extent topological and spectral aspects (global scale) as well as key constituents (local scale) of functional networks carry the same information as structural networks.

On the global scale, we observed topological aspects of functional networks—clustering, properties of shortest paths, and assortativity—to strongly deviate from ground truth. Possible information about the structural networks’ topological organization was largely obfuscated by different edge densities (complete vs. sparse networks) as well as by excessive clustering of vertices in functional networks. This deviation was mostly independent of properties of the coupling, of properties of the oscillators’ eigendynamics, and of uncertainties in deriving functional networks from data. We observed exceptions for assortativity of random coupling topologies but these were to be expected. Problematically, topological aspects have been repeatedly used to characterize natural and man-made systems in the past^68–73^ and have already been shown to be sensitive to a number of influencing factors, such as, e.g., constraints on the spatial^95–97^ and temporal^98^ sampling of a system.

Spectral aspects based on Laplacian matrices of functional networks, on the other hand, often conformed to the ones of structural networks when taking the matrices’ full eigenvalue spectra into account. Yet, typically, only synchronizability (the ratio of largest and smallest non-vanishing eigenvalue) is investigated^99–101^ due to its direct interpretation as an indicator for the stability of a networked dynamical system’s synchronized state^89, 90^. Synchronizability of functional networks, however, was only rarely similar to the ones of structural networks. Future research might also consider the often disregarded full eigenvalue spectrum of the Laplacian matrix to provide deeper insights into the dynamics of a complex system^102^.

On the local scale, we observed aspects of functional networks to frequently conform to ground truth. This was particularly true for key constituents, namely most central vertices and edges. The contribution of vertices to the networks’ shortest paths were similar between functional and structural networks (significant rank correlation between betweenness centrality values) for weak to intermediate coupling strengths. We also observed rankings of eigenvector centralities for vertices and edges to be correlated for an even wider range of coupling strengths. These relationships point to a backbone-like structure in the functional networks which resembles the structural networks to a high degree. However, congruencies differed strongly for different coupling topologies: the more narrow the degree distribution of the structural networks (most narrow for small-world topologies and widest for scale-free topologies), the weaker was the observed congruence. Large uncertainties in deriving functional networks from data lessened these congruencies.

Besides coupling strength and coupling topology, other influencing factors impacted only weakly on possible relationships between global and local scale aspects of functional networks and ground truth. Concerning the estimator for the strength of interaction to derive functional networks, relationships were, on average, slightly weaker for mutual information in comparison to those for maximum-lag cross correlation. This minor difference can most probably be related to the only weak non-linearity and (linear) diffusive couplings of the employed oscillators for which the estimators have different sensitivities^77^. Consequently, congruencies between structural and functional networks might also differ with other types of oscillators, but in general we expect similar findings^77^.

Regarding network size, there was a tendency for local aspects of smaller functional networks to conform more strongly to ground truth than larger networks. On the one hand, this might be related to the fact that for smaller networks there are less network constituents and, consequently, fewer chances to falsely assign centrality ranks—or to misidentify a key constituent—in functional networks. On the other hand, we introduced more (less) couplings between oscillators with larger (smaller) network sizes since we kept the edge density in the structural networks constant. Given our choice of other control parameters of the couplings, this might result in different dynamics among oscillators. Together, this hinders a thorough evaluation of the impact of the network size on the extent to which local aspects of the organization of a structural network can be revealed with the functional network ansatz. Such an evaluation would need to be carried out in future studies.

Summarizing our findings, local aspects of the organization of structural networks—representing complex dynamical systems—could be revealed from interactions estimated from time series of the systems’ dynamics to a large extent for various conditions, while global aspects could not. A comparison of our findings with those from related studies^29–63^ is only possible to a limited degree since studies largely focused on identifying edges of structural networks. Given that many of theses studies reported non-perfect degrees of success in this task, it is to be expected that functional and structural networks do not coincide in all aspects. Indeed, our results for the global scale corroborate this expectation. Surprisingly, however, local aspects—such as the contribution of vertices to shortest paths and the ranking of highest-rated network constituents—still showed high accordances between functional and structural networks. Detailed knowledge about such local aspects might be more informative than global aspects, particularly for field-data analysis.

We conjecture that further improvements can be achieved with surrogate approaches for functional networks. While such approaches are typically based on the preservation of degree or strength distributions of networks^103–110^,future development of surrogate networks designed to preserve more complex aspects of networks (such as the constituents’ contributions to shortest paths and the eigenspectra of the networks’ Laplacian matrices^111^) might substantially improve the characterization of a structural network’s organization. Similarly, further improvements might be achieved by refining time-series-analysis techniques. While there are techniques designed to weaken the adverse effect of indirect interactions (e.g., excessive clustering of vertices or transitivity) in characterizing properties of pairwise interactions^112–120^, their effectiveness was reported to be limited for larger systems^121^. Recent research into, e.g., higher-order interactions^122–124^ and dynamical interaction mechanisms^125, 126^ might stimulate the development of more appropriate time-series-analysis techniques.

With our investigations, we evaluated how well local and global aspects of structural networks can be revealed from properties of interactions, which are derived from time series data. By considering the mesocopic scale^127–133^ as well as time-dependent changes of networks^134–136^, future studies could add to our understanding of the relationship between structure and function of complex dynamical systems.

## Methods

### Oscillator networks

We generate time series of observables from *V* Rössler oscillators^137, 138^ diffusively coupled onto networks with complex coupling topologies

2 $$\begin{aligned} {\dot{x}}_i(t)= & {} \omega _i y_i-z_i+\frac{\varepsilon }{\langle \nu \rangle } \sum _{j\ne i}^V{\mathscr{A}} _{ij}(x_{j}-x_i) \nonumber \\ {\dot{y}}_i(t)= & {} \omega _i x_i + 0.1y_i \nonumber \\ {\dot{z}}_i(t)= & {} 0.1 + z_i(x_i-18.0), \end{aligned}$$

where the oscillators’ natural frequencies $\omega _i$ are drawn from a normal distribution ${\mathscr{N}}(1, \Delta \omega )$ with a mean of 1 and standard deviation $\Delta \omega$. For reference, $\Delta \omega$ is termed frequency inhomogeneity. Control parameters are chosen so that the Rössler oscillators exhibit chaotic motion if uncoupled. We increase global coupling strength $\varepsilon$ from 0 to 0.01 in steps of 0.0002 and frequency inhomogeneity $\Delta \omega$ from 0.025 to 0.2 in steps of 0.025. ${\mathscr{A}} \in \left\{ 0,1\right\} ^{V \times V}$ denotes the adjacency matrix representing the structural network. ${\mathscr{A}} _{ij}=1$ if and only if oscillators *i* and *j* are coupled, and 0 otherwise. On average, each oscillator is directly connected to $\langle \nu \rangle =\frac{1}{V}\sum _{i,j}^V{\mathscr{A}}_{ij}$ oscillators ($\langle \nu \rangle$ denotes the mean degree). For our investigations, $\langle \nu \rangle =8$ for networks of $V=50$ vertices and $E=400$ edges. For other sizes ($V=25, V=100$), we keep the edges density constant (i.e., $\langle \nu \rangle /(V-1)={{\text{const.}}}$) and change the range of coupling strengths while keeping the range of frequency inhomogeneity. For $V=25$, we increase $\varepsilon$ from 0 to 0.04 in steps of 0.0008, and for $V=100$, we increased $\varepsilon$ from 0 to 0.0025 in steps of 0.00005.

Coupling topologies are of

- random type^139^; probability for an edge to exist $p_{{\text{r}}} = 0.033$. We require $E = \langle \nu \rangle V/2$;
- scale-free type^140^; preferential attachment with initial number of vertices $m_0=\langle \nu \rangle + 1$ and growth parameter of $m=\langle \nu \rangle / 2$;
- small-world type^141^; each vertex is connected to $\langle \nu \rangle$ nearest neighbors with cyclic boundary conditions; edge rewiring with probability $p_{{\text{sw}}} = 0.1$.

With initial condition near the attractor, Eq. (2) is integrated with the Runge-Kutta-Fehlberg method with an adaptive step size and a sampling interval of 1. After discarding $2\cdot 10^4$ transients, we collect time series $u _i(t)$, $i=1,\ldots ,V$, of the *x*-component of oscillators for $t=1,\ldots , T$, where $T=2\cdot 10^4$ is the number of time steps. Time series contain at least 2000 oscillations of the slowest oscillator.

### Estimators for strength of interactions

**Maximum-lag cross correlation.** The (normalized) maximum-lag cross-correlation function between two normalized (zero mean and unit variance) time series $u _i(t)$ and $u _j(t)$ can be defined as^77^

3 $$\begin{aligned} \sigma _{ij} = \max _{\tau } \left\{ \left| \frac{\xi (u _i,u _j)(\tau )}{\sqrt{\xi (u _i, u _i)(0)\xi (u _j,u _j)(0)}} \right| \right\} , \end{aligned}$$

using the linear cross-correlation function

4 $$\begin{aligned} \xi (u _i,u _j)(\tau ) = {\left\{ \begin{array}{ll} \sum _{t=1}^{T-\tau } u _i(t+\tau ) u _j(t), &{} \tau \ge 0 \\ \xi (u _i,u _j)(-\tau ), &{} \tau < 0\text{,} \end{array}\right. } \end{aligned}$$

and where $\tau$ denotes the time lag. $\sigma _{ij}$ is confined to the interval [0, 1] with high values indicating that the two time series have a similar course in time (though possibly shifted by $\tau$) while dissimilar time series will result in values close to zero.

**Mutual information.** An estimator for the mutual information between two normalized (zero mean and unit variance) time series $u _i(t)$ and $u _j(t)$

5 $$\begin{aligned} \mu _{ij}=\sum _{k'\in u _i}\sum _{l'\in u _j} p_{(u _i,u _j)}(k',l') \log \frac{p_{(u _i,u _j)}(k',l')}{p_{u _i} (k')p_{u _j}(l')}, \end{aligned}$$

which is based on equidistant binning, can be defined as^76, 77^

6 $$\begin{aligned} \mu _{ij} =\log (T)+(1/T)\sum _{k=1}^Q\sum _{l=1}^Q \kappa _{k,l} \log \frac{\kappa _{k,l}}{\kappa _{k}\kappa _{l}}, \end{aligned}$$

where $\kappa _{k}$ ($\kappa _{l}$) approximates the probability $p_{u _i}(k')$ ($p_{u _j}(l')$) by the relative frequencies of occurrence of values of $u _i$ ($u _j$) in bin *k* (*l*) of the equidistantly partitioned range of $u _i$ ($u _l$). Similarly, $\kappa _{k,l}$ approximates the joint probability $p_{(u _i,u _j)}(k',l')$. We normalize $\mu _{ij}$ with respect to the maximum value that can be achieved for identical systems and given the time series’ length *T* and the number of bins *Q* (we here use $Q=40$ bins). $\mu _{ij}$ is thus confined to the interval [0, 1] with high values indicating that the two time series have a similar course in time while dissimilar time series will result in values close to zero.

### Global network aspects

In the following, the subscript ${{\text{s}}}$ resp. ${{\text{f}}}$ indicates measures used for our (binary) structural networks resp. (weighted) functional networks.

**Global clustering coefficient.** The global clustering coefficient measures the tendency of vertices to cluster together. For binary networks consisting of *V* vertices, we follow Ref.^141^ and define the global clustering coefficient as:

$$\begin{aligned} C_{{\text{s}}} = \frac{1}{V} \sum _i^V \frac{2 U(i)}{\nu _i (\nu _i-1)}, \end{aligned}$$

where *U*(*i*) is the number of triangles of three mutually connected vertices including vertex *i*, and $\nu _i$ is the degree of vertex *i* ($\nu _i=\sum _{j=1}^V{\mathscr{A}} _{ij}$, where ${\mathscr{A}} _{ij}$ are elements of the adjacency matrix).

Following Refs.^142^ and^104^, the global clustering coefficient of a weighted network can be defined as:

$$\begin{aligned} C'_{{\text{f}}} = \left( {\begin{array}{c}V\\ 3\end{array}}\right) ^{-1} \sum _{i=1}^{N}\sum _{j=1}^{i-1}\sum _{k=1}^{j-1} \frac{\root 3 \of {{\mathscr{W}} _{ij} {\mathscr{W}} _{jk} {\mathscr{W}} _{ki}}}{\max ({\mathscr{W}})}, \end{aligned}$$

where ${\mathscr{W}}$ is the weight matrix of the network, whose entries ${\mathscr{W}} _{ij}$ are the edge weights.

To further facilitate the comparison between the binary structural networks and the weighted functional networks, we also normalize the global clustering coefficient $C'_{{\text{f}}}$ for weighted networks by the mean edge weight ${\overline{{\mathscr{W}}}} = \frac{2}{V(V-1)}\sum _{i}^V \sum _{j=i+1}^V{\mathscr{W}} _{ij}$:

$$\begin{aligned} C_{{\text{f}}} = \frac{C'_{{\text{f}}}}{{\overline{{\mathscr{W}}}}}. \end{aligned}$$

**Average shortest path length.** The average shortest path length measures the typical separation between two vertices. For binary networks, the length $\psi _{ij}$ of a shortest path between a pair of vertices (*i*, *j*) is the minimum number of edges that have to be traversed to reach vertex *j* when starting at vertex *i*. The average shortest path length can then be defined as:

$$\begin{aligned} L_{{\text{s}}} = \frac{2}{V(V-1)}\sum _{i}^V \sum _{j}^{i-1} \psi _{ij}. \end{aligned}$$

For a weighted network, one can define the length of an edge as the inverse of the weight of that edge. If we exclude the path from one vertex to itself from the mean, the average shortest path length can be defined as^104^:

$$\begin{aligned} L'_{{\text{f}}} = \left( {\begin{array}{c}N\\ 2\end{array}}\right) ^{-1} \sum _{i=1}^V \sum _{j=1}^{i-1}\min _l \min _{P\in {\mathscr{P}}_{ij}^l}\sum _{k=1}^{l-1}{\mathscr{W}} _{P_{k}P_{k+1}}^{-1}, \end{aligned}$$

where ${\mathscr{P}}_{ij}^l :=\Big \{ P\in \{1,\dots ,V \}^l \Big | P_1=i, P_l=j \Big \}$ is the set of all paths that traverse *l* edges from vertex *i* to *j* and ${\mathscr{W}} _{ij}^{-1}=\infty$ if and only if ${\mathscr{W}} _{ij}=0$.

To further facilitate the comparison between the binary structural networks and the weighted functional networks, we scale the average shortest path length $L'$ for weighted networks by the mean edge weight:

$$\begin{aligned} L_{{\text{f}}} = L'_{{\text{f}}} \, {\overline{{\mathscr{W}}}} \end{aligned}$$

**Assortativity.** Assortativity *A* quantifies whether vertices preferentially connect to vertices with a similar degree for binary networks^87, 88^ or with a similar strength for weighted networks. For binary networks, *A* is defined as the correlation coefficient over all pairs of degrees $\{(\nu _i, \nu _j)|{\mathscr{A}} _{ij}=1, 1\le (i,j)\le V\}$ of the vertices:

$$\begin{aligned} A_{{\text{s}}} = \left( 2 D_{1} \sum _{i=1}^{V} \sum _{j=1}^{i-1} {\mathscr{A}} _{ij} \nu _{i} \nu _{j} - \nu _{2}^{2} \right) / D_1 D_3 - D_2^2, \end{aligned}$$

with $D_m = \sum _{i=1}^{V}\nu _i^m$ and $\nu _i$ is the vertex degree of vertex *i*.

For weighted networks, we follow Ref.^143^ and define *A* as the correlation coefficient over all pairs of strengths $\{(s _i, s _j), 1\le (i,j)\le V\}$ of the vertices:

$$\begin{aligned} A_{{\text{f}}} = \frac{\sum _{k=1}^{V(V-1)/2} (\zeta _k - {\overline{\zeta }}) (\zeta '_k - \overline{\zeta '})}{\sqrt{\sum _{k=1}^{V(V-1)/2} (\zeta _k - {\overline{\zeta }})}\sqrt{\sum _{k=1}^{V(V-1)/2} (\zeta '_k - \overline{\zeta '})}}, \end{aligned}$$

where $\zeta _k$ ($\zeta '_k$) is the *k*-th element of the collection of the first (second) entry of all ordered pair of vertex strengths $\{(s _i, s _j)| 1 \le (i,j) \le V\}$. The strength of vertex *i* is defined as $s _i = \sum _{j}^V{\mathscr{W}} _{ij}$. ${\overline{\zeta }}$ and $\overline{\zeta '}$ are the collection’s respective means. Positive (negative) values of $A_{{{\text{s}}}}$ resp. of $A_{{{\text{f}}}}$ indicate an assortative (disassortative) network.

**Synchronizability.** The stability of the globally synchronized state of a network of coupled oscillators can be characterized by the *eigenratio* $S = \lambda _V/\lambda _2$^89, 90^. $\lambda _V$ denotes the largest eigenvalue of the Laplacian matrix ${\mathscr{L}}$ of the network (${\mathscr{L}}_{ij} = \nu _i \delta _{ij} - {\mathscr{A}} _{ij}$ for binary networks and ${\mathscr{L}}_{ij} = s_i \delta _{ij} - {\mathscr{W}} _{ij}$ for weighted networks^90, 144^, where $\delta$ is the Kronecker delta and $s_i$ denotes the strength of vertex *i*). $\lambda _2$ denotes the second smallest eigenvalue of the Laplacian (the smallest being 0). Given some vertex dynamics, the higher *S* the less stable is the synchronized state of the network. This interpretation crucially depends on the definition of *S* (note that other definitions can be found in the literature, e.g., $S = \lambda _2/\lambda _V$).

### Centralities of vertices and edges

For our investigations, we utilize the concepts of betweenness and eigenvector centrality since corresponding centrality measures are available for both vertices and edges^133^. Betweenness centrality is based on shortest paths, which requires the definition of “length” $\psi _{ij}$ of a path between vertices *i* and *j* or between edges *i* and *j*. The length $\psi _{ij}$ of a shortest path P between vertices *i* and *j* in a binary network is the number of edges along this path, and we utilize the same definition for the length $\psi _{ij}$ of P between edges *i* and *j*. For *i* and *j* being connected to a same vertex/edge, we define $\psi _{ij} :=0$. In case of a weighted network, we relate the length $\psi _{ij}$ of P between vertices/edges *i* and *j* to the sum of the inverse weights of edges along this path. In case of adjacent edges, i.e., edges connected by a single vertex, we again define $\psi _{ij} :=0$. We denote the set of *V* vertices (*E* edges) in a network as ${\mathscr{V}}$ (${\mathscr{E}}$).

**Betweenness centrality,** Betweenness centrality ${\mathscr{C}}^{\mathrm{B}}$ highlights a constituent as central if it acts as bottleneck in a network. Vertex betweenness centrality (of vertex *i*) can be defined as^145–148^

7 $$\begin{aligned} {\mathscr{C}}^{\mathrm{B}}_{\mathrm{v}}(i)=\frac{2}{(V-1)(V-2)}\sum _{i\ne j\ne k}\frac{q_{jk}(i)}{G_{jk}}, \end{aligned}$$

where $\left\{ i,j,k\right\} \in {\mathscr{V}}$, and $q_{jk}(i)$ is the number of shortest paths between vertices *j* and *k* running through vertex *i*. $G_{jk}$ is the total number of shortest paths between vertices *j* and *k*.

Edge betweenness centrality (of edge *i*) can be defined as^149, 150^

8 $$\begin{aligned} {\mathscr{C}}^{\mathrm{B}}_{\mathrm{e}}(i)=\frac{2}{V(V-1)}\sum _{j\ne k}\frac{q_{jk}(i)}{G_{jk}}, \end{aligned}$$

where $i\in {\mathscr{E}}$, $\left\{ j,k\right\} \in {\mathscr{V}}$, $q_{jk}(i)$ is the number of shortest paths between vertices *j* and *k* running through edge *i*, and $G_{jk}$ is the total number of shortest paths between vertices *j* and *k*.

**Eigenvector centrality.** Eigenvector centrality ${\mathscr{C}}^{\mathrm{E}}$ is a degree-/strength-based concept and this centrality highlights a constituent as central if it is connected to other central constituents. Vertex eigenvector centrality (of vertex *i*) is defined as the *i*-th entry of the eigenvector $\vec {v}$ corresponding to the dominant eigenvalue $\lambda '_{\max }$ of matrix $\Xi$^151^, which we derive from the eigenvector equation $\Xi \vec {v}=\lambda '\vec {v}$ using the power iteration method

9 $$\begin{aligned} {\mathscr{C}}^{\mathrm{E}}_{\mathrm{v}}(i)=\frac{1}{\lambda '_{\max }}\sum _{j}^{}\Xi _{ij}\,{\mathscr{C}}^{\mathrm{E}}_{\mathrm{v}}(j), \end{aligned}$$

with $\left\{ k,l\right\} \in {\mathscr{V}}$. Here, $\Xi$ denotes the adjacency matrix ${\mathscr{A}} \in \left\{ 0,1\right\} ^{V \times V}$ of a binary network, with ${\mathscr{A}} _{ij}=1$ if there is an edge between vertices *i* and *j*, and 0 otherwise. In case of a weighted network, $\Xi$ denotes the weight matrix ${\mathscr{W}} \in {\mathbb {R}}_+^{V \times V}$, with ${\mathscr{W}} _{ij}$ denoting the weight of an edge between vertices *i* and *j*. We define ${\mathscr{A}} _{ii} :=0 \,\forall \, i$ and ${\mathscr{W}} _{ii} :=0 \,\forall \, i$ with $i\in {\mathscr{V}}$ to exclude self-loops.

Edge eigenvector centrality (of edge *i*) is defined as^133^

10 $$\begin{aligned} {\mathscr{C}}^{\mathrm{E}}_{\mathrm{e}}(i)=\frac{1}{\lambda '_{\max }}\sum _{j}^{}\Xi '_{ij}\,{\mathscr{C}}^{\mathrm{E}}_{\mathrm{e}}(j), \end{aligned}$$

with $\left\{ i,j\right\} \in {\mathscr{E}}$. Here, $\Xi '$ denotes the edge adjacency matrix ${\mathscr{A}} ^{\mathrm{(e)}} \in \left\{ 0,1\right\} ^{E \times E}$ of a binary network, with ${\mathscr{A}} ^{\mathrm{(e)}}_{ij}=1$ if edges *i* and *j* are connected to a same vertex, and 0 otherwise. In case of a weighted network, $\Xi '$ denotes the edge weight matrix ${\mathscr{W}} ^{\mathrm{(e)}} \in {\mathbb {R}}_+^{E \times E}$ whose entries ${\mathscr{W}} ^{\mathrm{(e)}}_{ij}$ are assigned the average weight of edges *i* and *j* if these edges are connected to a same vertex, and 0 otherwise. As above, we define ${\mathscr{A}} ^{\mathrm{(e)}}_{ii} :=0 \,\forall \, i$ and ${\mathscr{W}} ^{\mathrm{(e)}}_{ii} :=0 \,\forall \, i$ with $i\in {\mathscr{E}}$.

### Miscellaneous

The following table reports the number of pairs of structural and functional networks for each type of coupling topology, regime of coupling strengths (I, II, and III), and estimator of strength of interaction ($\sigma$ and $\mu$).

**Table:** 

	$\sigma$			$\mu$		
	I	II	III	I	II	III
Random	1671	2769	1910	3159	2668	523
Scale-free	1984	2427	1722	3838	1563	732
Small-world	1808	1889	2090	2531	2055	1201
Empirical	117	294	0	64	347	0

## Data Availability

The original contributions presented in the study are included in the article, further inquiries can be directed to the corresponding author.
